# Bidirectional remodeling of the central auditory system caused by unilateral auditory deprivation

**DOI:** 10.3389/fneur.2024.1414738

**Published:** 2024-07-16

**Authors:** Xinying Ge, Cong Xu, Jinsheng Dai, Mo Zhou, Jinfeng Liu, Ningyu Wang

**Affiliations:** Department of Otorhinolaryngology Head and Neck Surgery, Beijing Chaoyang Hospital, Capital Medical University, Beijing, China

**Keywords:** unilateral auditory deprivation, neural plasticity, single-sided deafness, bidirectional remodeling, central auditory system

## Abstract

Unilateral auditory deprivation (UAD) results in cross-modal reorganization of the auditory cortex (AC), which can impair auditory and cognitive functions and diminish the recovery effect of cochlear implantation. Moreover, the subcortical areas provide extensive ascending projections to the AC. To date, a thorough systematic study of subcortical auditory neural plasticity has not been undertaken. Therefore, this review aims to summarize the current evidence on the bidirectional remodeling of the central auditory system caused by UAD, particularly the changes in subcortical neural plasticity. Lateral changes occur in the cochlear nucleus, lateral superior olive, medial nucleus of the trapezoid body, inferior colliculus, and AC of individuals with UAD. Moreover, asymmetric neural activity becomes less prominent in the higher auditory nuclei, which may be due to cross-projection regulation of the bilateral pathway. As a result, subcortical auditory neural plasticity caused by UAD may contribute to the outcomes of cochlear implantation in patients with single-sided deafness (SSD), and the development of intervention strategies for patients with SSD is crucial. Considering that previous studies have focused predominantly on the neural plasticity of the AC, we believe that bidirectional remodeling of subcortical areas after UAD is also crucial for investigating the mechanisms of interventions.

## 1 Introduction

Single-sided deafness (SSD) refers to normal hearing in one ear and profound sensorineural hearing loss in the contralateral ear (1, 2). The prevalence of SSD in neonates is reported to be 0.1%, and the estimated prevalence in school-aged children is approximately 14% (3). Compared to bilateral hearing loss, unilateral hearing loss is more likely to be ignored (mainly because the opposite ear can be used to communicate) without timely intervention, thereby compromising speech and language development in children (4).

Binaural hearing is essential for normal auditory processing. The balance of bilateral input is also key to binaural integrated coding. The interaural time difference and the interaural level difference signals are the main cues that contribute to sound localization (5). Access to these cues is insufficient in patients with SSD, leading to a reduction in sound localization and speech recognition scores (6). Monaural input has an extensive impact on the development of brain networks related to higher-order cognitive function (1). SSD in children further affects language learning, cognitive ability, and academic performance, which are closely associated with a lack of spatial abilities and binaural hearing (7).

At present, one of the main challenges in binaural hearing rehabilitation is appropriate intervention for congenital or early development of SSD. Based on clinical observations, recent studies have shown that contralateral routing of signals from hearing aids, bone-conduction devices, and cochlear implants (CI) can improve sound localization and speech recognition scores (1, 8–10). However, rehabilitation outcomes remain suboptimal in some patients, characterized by poor performance in speech audiometry and sound localization measurements (11–13). Lacking auditory input due to SSD during the sensitive period leads to a series of adaptive structural and functional changes in the central auditory system (CAS), known as unilateral auditory deprivation (UAD) (8, 14, 15). For children with UAD after CI, one concern is that they may not benefit from the CI. When one sensory system fails, the brain will undergo a series of adaptive structural and functional changes to reorganize its neural network, which is known as remodeling (16–18). Once the remodeling in the CAS caused by UAD becomes stable, it is difficult to reverse and may diminish the recovery effect of CI (6, 7). Therefore, clarifying the remodeling of the CAS after UAD is important for achieving effective interventions.

Previous studies have described the remodeling of the auditory cortex (AC) after UAD (19–21); however, studies on the remodeling of subcortical nuclei are rare. This overview aims to summarize the current evidence on the structural and functional bidirectional remodeling of the subcortical nuclei and AC caused by UAD.

## 2 Unilateral auditory deprivation

In the early stages of development, the CAS has a remarkable ability to adjust to an ever-changing environment and optimize sensory acuity through experience-dependent mechanisms (22). This ability is referred to as plasticity (23–25). During the early stages of auditory development, the CAS remains more susceptible to alterations by an experience called sensitive periods (26, 27). Some of these sensitive periods are called critical: the absence of certain juvenile experiences cannot be fully compensated for later in life (26, 28). Moreover, the sensitive period of auditory development is also the critical period of auditory rehabilitation after deafness (29, 30), and the plasticity of the auditory center will be significantly decreased at the end of the critical period; sensitive periods end (become critical) as too many synapses that depend on sensory input are eventually eliminated, compromising the computational power of the corresponding neuronal networks (22, 31). Although critical periods are different based on the individual, found through animal experiments and clinical observations, it should be at approximately 3.5 years in humans (7, 31–34).

Single-sided deafness is diagnosed based on the degree of unilateral hearing loss. However, UAD is diagnosed based on the status of the CAS, and specifically the impact on the CAS after a long period of SSD. Auditory deprivation in early development prevents functional maturation, delays cortical synaptogenesis, and increases subsequent synaptic elimination, which leads to the weakening or loss of auditory information processing ability in the CAS (22), including intensity coding, cortical column functioning, cochleotopic representation, representation of auditory space, and corticocortical interactions (26).

Moreover, the degree of UAD can be attributed to individual differences in onset time, as well as the degree and duration of SSD (35). For example, unilateral conductive hearing loss in young rats causes stronger lateralization of the primary AC than in adult rats (36). The effect of auditory deprivation on adults with post-lingual deafness is small (37). Therefore, particular emphasis is placed on the importance of the auditory experience in the early sensitive periods of development. Even brief early hearing experiences can have extensive developmental effects and may prevent deprivation-induced developmental deficits (7).

The degree of UAD is also related to the timeliness and effectiveness of SSD treatment (6, 38). The outcomes of CI depend on the developmental period during which implantation occurs. Developmental delays are quickly compensated for (within a year of CI use) in children who are implanted earlier, within the first 3.5 years of life (39, 40). However, without intervention, the effect of SSD on the CAS eventually stabilizes, representing the degree of UAD. This degree reflects the level of central compensation and can predict the level of difficulty and effect of rehabilitation. The age of intervention in SSD patients (11), intervention strategies (22), and auditory training after intervention (6, 41) are crucial to determine the effectiveness of hearing restoration. Liu et al. (6) reported that UAD can distort tonotopic maps, disrupt binaural integration (7, 8), reorganize neural networks, and change synaptic transmission in the primary AC or sub-cortex (6, 22). UAD eventually results in unique bidirectional remodeling of the CAS and impaired processing of binaural cues (7).

## 3 Bidirectional remodeling of the CAS caused by UAD

The development and perfection of the auditory system and the formation of normal auditory function require symmetrical or balanced pathways on both sides of the auditory pathway to establish normal pathways and synaptic connections and finally form a complex connectome. A connectome is a network map of effective synaptic connections and neural projections in the nervous system. It includes complex network connectors, such as vision-hearing, motion-hearing, and spatial location-hearing (22).

Congenital or early development of SSD results in the loss or reduction of auditory transmission on one side, which further affects the individual wiring and coupling patterns of the brain, leading to the formation of an abnormal connectome and obvious bidirectional remodeling. Degenerative changes occur on the affected side of the CAS, whereas adaptation is enhanced on the contralateral side (6). Accordingly, evidence of cross-modal reorganization of the AC has been observed in deaf individuals (42, 43), and such bias may result in stronger coupling to the remaining sensory systems and reorganization within the affected sensory system (22).

Only if SSD appears in the early developmental stage, and without effective intervention, is it difficult to reverse the remodeling of the CAS. While auditory deprivation does occur in the mature CAS and central remodeling still exists, it is significantly reduced. The reorganization of aural preference is present only in animals with early-onset asymmetrical hearing (7). Studies have shown that after the age of 4 months, long-term use of a single-sided CI did not induce a significant change in aural preference, thereby demonstrating a critical developmental period for the reorganization of aural preference in the primary AC (26, 31). This indicates that abnormal aural preference develops only if unilateral hearing occurs early in life. The younger the subject with SSD, the stronger the plasticity of the CAS, which results in more obvious degeneration of the affected side and adaptive enhancement of the contralateral side of the auditory pathway (44, 45). Moreover, a longer SSD duration can lead to more obvious degeneration of the affected side and adaptive enhancement of the contralateral (6). Auditory preference only emphasizes the enhancement of the contralateral pathway and ignores the weakening of the deaf side, whereas bidirectional preference is more appropriate.

### 3.1 Bidirectional remodeling of the AC caused by UAD

The primary AC receives input from both sides. This means that even complete deafness in one ear does not completely deprive the cortical neurons of input. Cross-modal recombination in the AC differs between the healthy and affected sides. The strongest reorganization is measured in the hemisphere ipsilateral to the hearing ear; this cortex undergoes extensive reorganization with respect to both response amplitudes and latencies (31). For example, the local field potential amplitudes are larger for stimulation of the ipsilateral ear, and the latencies are shorter for stimulation of the ipsilateral ear. However, such results are never observed in normal-hearing animals or animals with congenital binaural deafness (26, 31). These observations imply a change in the aural preference of the cortical area ipsilateral to the hearing ear and an extensive change in the representation of the auditory space. Kral et al. (31) demonstrated unilateral aural preference caused by SSD within an early sensitive period and pronounced and rapid reorganization in the primary AC. A follow-up study investigated the representation of both ears on individual neurons; the representation of the hearing ear was strengthened, and that of the deaf ear was weakened (46). Once the AC is reorganized, neuronal resources on the weak side are diverted to visual or somatosensory processing, potentially affecting performance after hearing restoration (30, 47). Growing evidence suggests that poor outcomes in patients with a CI may be explained by cross-modal reorganization, whereby a sensory modality (i.e., vision and somatosensation) may recruit another sensory system (i.e., audition) to compensate for deficits in the deprived modality (30).

Gordon et al. (48) showed that over-strengthening of the neural projections and AC corresponding to unilateral stimulation results in an abnormally large contralateral lateralization of activity. Sandmann et al. (49) used electroencephalography to examine cross-modal reorganization in the AC of post-lingually deafened CI users. Their results confirmed a visual take-over in the AC of CI users. An incomplete reversal of this deafness-induced cortical reorganization might limit the clinical benefit of CI and help explain the high inter-subject variability in auditory speech comprehension. Encephalographic measures of activity in the left and right auditory areas of the brain confirmed that aural preference was established by depriving pathways from the deaf ear and allowing for the strengthening of input from the better ear (50, 51). Taken together, the abnormal laterality activity of the AC after SSD can reflect the degree of cortical remodeling and the severity of UAD.

In summary, data from animal models at both the local field and unit levels of the analysis show that UAD in early development promotes processes in the hemisphere ipsilateral to the hearing ear that are different from those in the contralateral hemisphere. Consistent with this, the representation of the hearing ear is strengthened, and that of the deaf ear is weakened in both hemispheres. Thus, the condition of SSD leads to the development of a “stronger ear” and a “weaker ear” (26, 31).

### 3.2 Bidirectional remodeling of the subcortical nuclei caused by UAD

Afferent decreases and asymmetry affect the ascending pathway. Theoretically, the effect in the lower nuclei is especially obvious during the uncrossed stage, whereas the influence of the SSD may be reduced in the case of cross nuclei. However, subcortical areas may be more skewed (52–55). This indicates that some of the reorganizations take place subcortically and are present during cortical input, while the effects of aural preference are even more extensively expressed in subsequent cortical processing.

The CN is located on both sides of the brainstem. It receives excitatory afferents from the auditory nerve and relays auditory information to higher levels of the auditory pathway. In rats suffering from unilateral cochlea removal at early development, cochlear hair cells are lost, followed by the degeneration of spiral ganglion neurons. This leads to impairments in both the transduction process in the inner ear and the transmission of auditory signals to the brain (28, 56). Consequently, morphological changes occur in the CN. According to Mostafapour et al. (57), cochlear resection in mice leads to 61% neuron loss in the ipsilateral anterior ventral cochlear nucleus (ACVN) on postnatal day 5 (P5) but less than 1% loss when resection is performed on P14. In contrast, Jakob et al. (56) found that the volume of the ipsilateral CN decreased significantly after P30, and there was no significant change on the contralateral side. This may be due to differences in the experimental animals or the different observation times. Moreover, Kim et al. (54) found that neural activity decreased in the ipsilateral CN after UAD, whereas aural dominance was increasingly attenuated at higher levels of the CAS, using Mn-enhanced magnetic resonance imaging. This difference may be due to the choice of adult experimental animals. These results indicate that, after UAD, the CN is first to receive the asymmetric influence of bilateral sound input, resulting in structural and even functional changes that are then transmitted to the next nucleus.

The superior olivary complex (SOC) is a major computational center in the auditory brainstem that mainly processes inter-aural-difference cues for sound localization. In all mammals, it comprises a group of auditory brainstem nuclei: the lateral superior olive (LSO), the medial superior olive (MSO), the trapezoid body, and peri-olivary nuclei (58–60).

**Figure 1:**
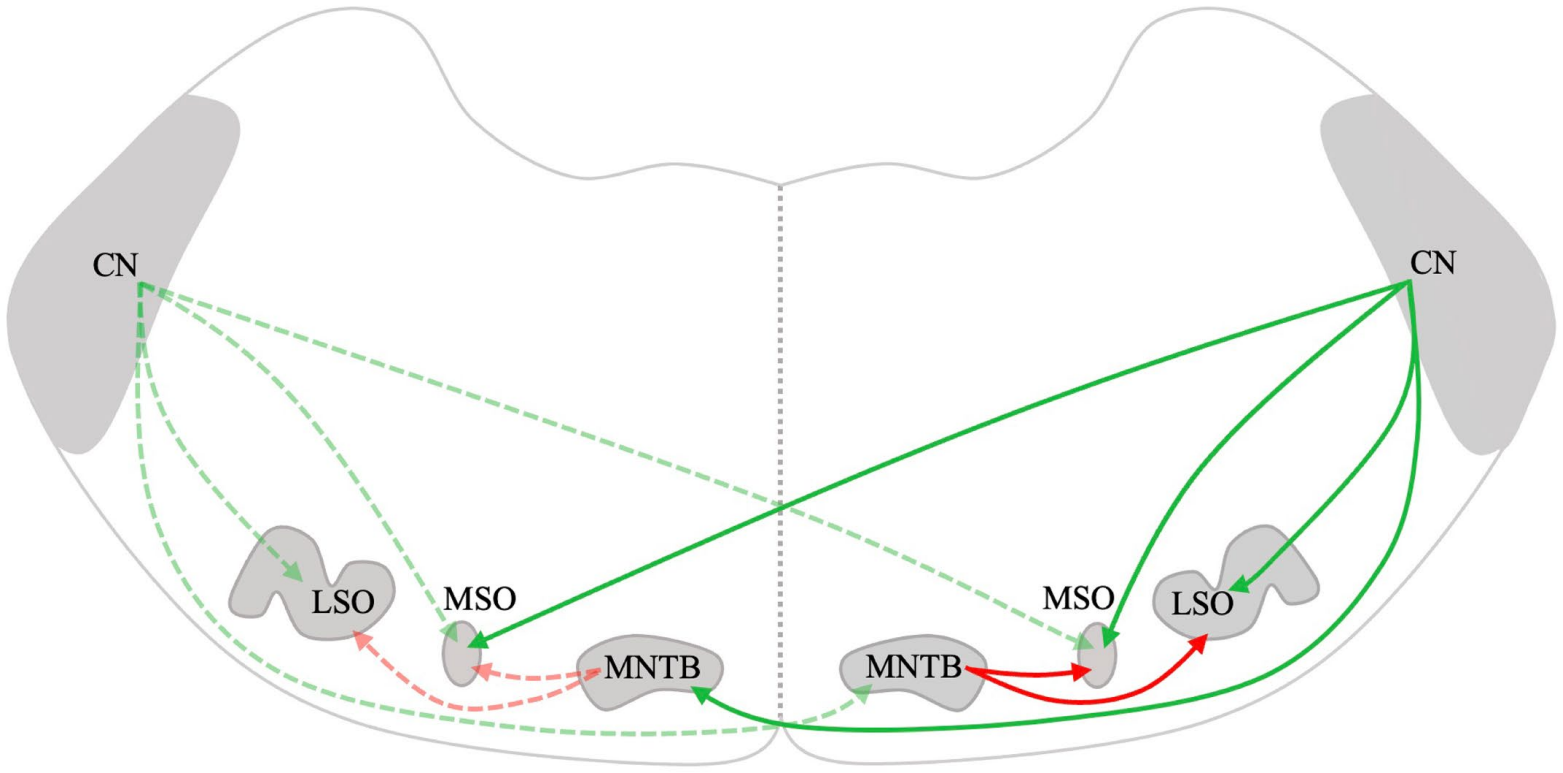
Schematic drawing of the mammalian lower auditory brainstem. Excitatory projections are depicted in green, while inhibitory projections are depicted in red. The dotted lines represent connections on the left and solid lines represent connections on the right. CN, Cochlear nucleus; LSO, Lateral superior olive; MSO, Medial superior olive; and MNTB, Medial nucleus of the trapezoid body.

Lateral superior olive neurons are excited by inputs from the ipsilateral AVCN and are inhibited by glycinergic neurons of the ipsilateral medial nucleus of the trapezoid body (MNTB) (Figure 1) (61). The LSO is the first level of the CAS where massive convergence of information from the two ears occurs. The LSO is mainly involved in the processing of interaural level differences and plays a major role in horizontal plane sound localization (5). Therefore, remodeling changes in the LSO after UAD are important. Zhou et al. (52) used *in vitro* whole-cell patch-clamp recordings to investigate the changes in electrophysiological activity in the LSO caused by UAD and found that UAD weakened the inhibitory driving force on the hearing side while strengthening the excitatory driving force on the ablated side. This indicates that asymmetric changes exist on both sides of the LSO after UAD and may lead to further disruption of binaural balance and interfere with the normal development of the LSO. Eventually, it may impede the rehabilitation of binaural hearing in congenital or early-development SSD.

The MSO lies midway through the SOC. It mainly receives excitatory afferent fibers from both ipsilateral and contralateral CN and inhibitory input from the MNTB and lateral nuclei of the trapezoid body (Figure 1) (62). The MSO primarily processes the interaural time difference and plays an important role in sound localization. Theoretically, UAD will have some effect on the structure and function of MSO. However, since the MSO receives bilateral projections, which are more complex than those in the LSO, it is possible that the bilateral difference in the MSO may not be significant. However, few studies have been conducted on the effects of UAD on the MSO. We will continue to pay attention to relevant research to further improve our understanding of the remodeling mechanism of CAS after UAD.

The MNTB receives excitatory inputs from the globular bushy cells of the contralateral CN (Figure 1) (63, 64). It is the main contributor to the inhibition of the sound localization pathway and projects to the MSO and LSO (53, 65, 66). Dai et al. (55) found increased GABA, Gly, and Glu in the ipsilateral MNTB at 4 weeks after cochlear ablation and increased GABAa-R, GABAb-R, Gly-R, and AMPA in the contralateral MNTB at 2 weeks in rats. Furthermore, Gly levels in the contralateral MNTB were significantly increased at 4 weeks (55). The distribution of neurotransmitters and receptors in the MNTB is asymmetrical after early-development SSD. Similarly, after unilateral cochlear ablation during early development in gerbils, the distribution of synaptic endings and neuronal architecture changed inconsistently on the lesioned side compared to that of the intact side of the MNTB (67). The lateralized changes in excitatory versus inhibitory transmitters and their receptors and the distribution of synaptic endings and neuronal architecture may account for the altered lateralization in activity in the auditory pathway following UAD.

The inferior colliculus (IC) is an important site for auditory conduction, relay, and integration. It accepts both the ascending and descending projections (68). Through whole-cell patch-clamp recordings, Vale et al. (69) found that synaptic inhibition was weakened in the ipsilateral IC after unilateral cochlear ablation, whereas commissural-evoked inhibitory synaptic conductance declined only in the contralateral IC of the ablated cochlea. Furthermore, they analyzed paired-pulse facilitation and found that inhibitory transmitter release was more affected in the ipsilateral IC (69). This indicates that excitability increases in the ipsilateral IC after UAD, which can be considered a compensatory effect. This phenomenon is similar to that observed in the LSO (52). Moreover, using Mn-enhanced magnetic resonance imaging, Kim et al. (54) found that neural activity decreased on both sides of the IC after UAD compared to normal mice. Although neural activity decreased on both sides of the IC due to UAD, the changes were asymmetrical and more pronounced on the contralateral side. These results indicate that although the IC receives bilateral auditory integration, UAD still leads to changes in neural activity in the IC, which eventually affects auditory integration in both ears.

**Figure 2:**
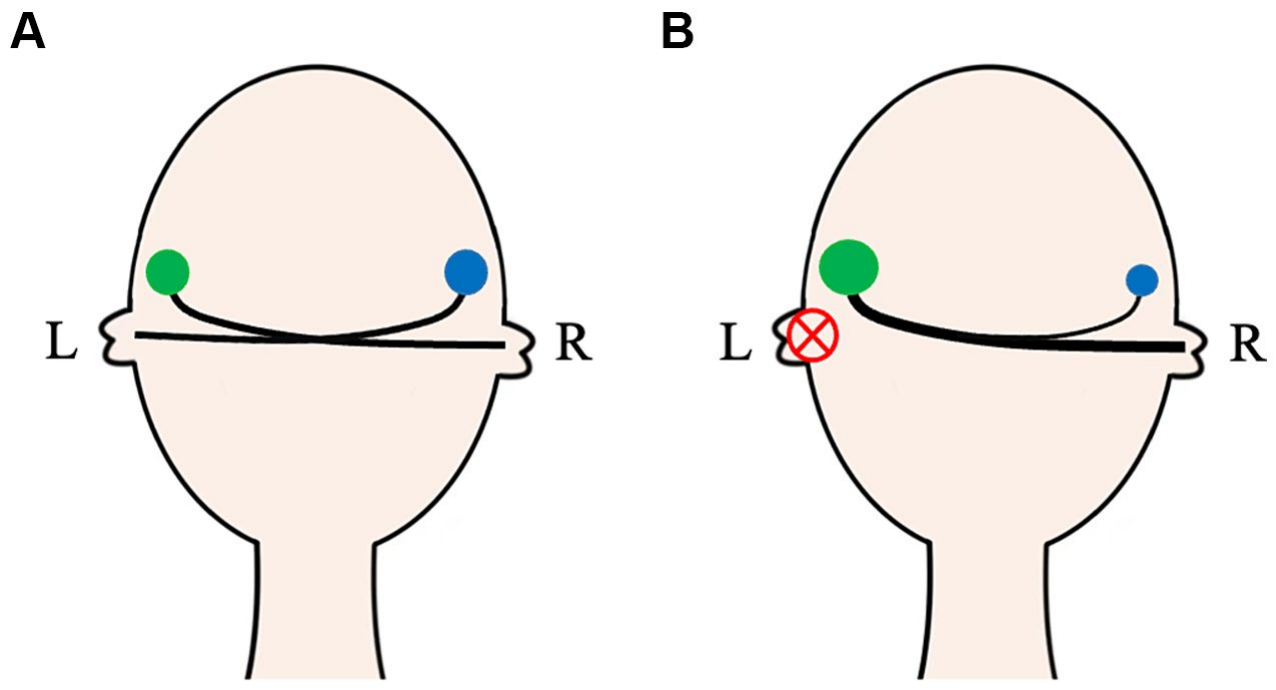
The auditory activity on both ears in normal and unilateral auditory deprivation patients. In normal-hearing people, the auditory activity of bilateral CASs (green and blue circles) is basically symmetrical **(A)**. For unilateral auditory deprivation patients, due to no effective sound afferents, bidirectional remodeling of the CAS occurs not only in the auditory nuclei but also in the auditory pathway **(B)**. CAS, Central auditory system; L, Left; R, Right.

Thus, UAD during early development leads to asymmetric changes in the CAS (Figure 2). By summarizing studies on the structural and functional remodeling of subcortical nuclei and the AC after UAD, we found that UAD during early development affects almost all nuclei in the CAS. The effect on the lower nuclei was evident, whereas the effect on the higher nuclei was weakened, which may be due to the cross-projection regulation of bilateral pathways. Nevertheless, the asymmetric changes in the CAS caused by UAD eventually lead to patients not using binaural dominance for accurate sound localization.

## 4 Remaining challenges

In summary, bidirectional remodeling of the AC and subcortical-related auditory nuclei caused by UAD can detrimentally impact both auditory and cognitive functions and may even diminish the positive effects of CI (6, 15, 22). However, the fundamental mechanisms and functional and structural changes in the bidirectional remodeling of the AC and subcortical-related auditory nuclei caused by UAD remain unclear. Even with the development of neural tracing technology, questions remain regarding genetic alterations, interactions between astrocytes or oligodendrocytes and neurons, changes in the electrical activities of neurons across various nuclei, and effects on neural circuits. Moreover, countering the limitations of hearing recovery caused by bidirectional remodeling and improving speech recognition and sound localization after UAD are pressing challenges that remain unresolved. Future research should focus on studying fundamental mechanisms of the bidirectional remodeling in the CAS caused by UAD and how to restore symmetrical bilateral hearing to improve spatial hearing abilities.

## Author contributions

XG: Conceptualization, Writing – original draft. CX: Funding acquisition, Conceptualization, Writing – original draft. JD: Writing – review & editing. MZ: Writing – review & editing. JL: Supervision, Writing – review & editing. NW: Funding acquisition, Supervision, Writing – review & editing.
